# Pseudotumeur cardiaque révélant une maladie de Behçet

**DOI:** 10.11604/pamj.2017.26.151.11478

**Published:** 2017-03-15

**Authors:** Fouad Nya, Abdessamad Abdou, Mehdi Bamous, Younes Moutakiallah, Noureddine Atmani, Aniss Seghrouchni, Mahdi Ait Houssa, Abdellatif Boulahya

**Affiliations:** 1 Service de Chirurgie Cardiaque, Hôpital Militaire d’Instruction Mohamed V, Faculté de Médecine et de Pharmacie, Université Mohamed V, Rabat, Maroc

**Keywords:** Pseudotumeur cardiaque, maladie de Behçet, Maroc, Cardiac pseudotumor, Behçet’s disease, Morocco

## Abstract

La thrombose intracardiaque est une complication rare de la maladie de Behçet (MB), qui peut se présenter comme une tumeur intracardiaque. Sa découverte précède, dans la moitié des cas, le diagnostic de MB. La mortalité élevée peut être en rapport avec des complications post-chirurgicales et/ou une atteinte associée des artères pulmonaires. Nous rapportons l’observation d’un jeune patient de 29 ans, aux antécédents d’aphtose bipolaire, qui a bénéficie d’une intervention chirurgicale après la découverte d’une tumeur de l’atrium et du ventricule droits. Il s’agissait d’un thrombus à l’examen anatomopathologique et dans les suites opératoires nous avons diagnostiqué une MB. L’évolution a été favorable sous traitement médical associant une corticothérapie, de la colchicine et des antivitamines K (AVK). La découverte d’une masse intracardiaque chez un sujet jeune doit faire évoquer le diagnostic de thrombus cardiaque et de maladie de Behçet, même en l’absence de facteur ethnique ou géographique prédisposant.

## Abstract (en)

Intracardiac thrombosis is a rare complication of Behçet’s disease (BD), which may manifest as intracardiac tumor. In half of cases, its detection precedes the diagnosis of MB. High mortality rates may be related to post-surgical complications and/or pulmonary arteries involvement. We report the case of a 29-year old young patient, with a previous history of bipolar aphthosis, who underwent surgery after the detection of right atrium and ventricle tumor. Anatomo-pathological examination showed thrombus and MB was diagnosed in the postoperative period. Patient’s evolution was favorable under medical treatment based on corticosteroids, colchicine and vitamin K antagonists (AVK). The detection of intracardiac mass in a young subject should suggest the diagnosis of cardiac thrombus and Behçet’s disease, even in the absence of ethnic or geographical risk factors.

## Introduction

La maladie de Behçet (MB) est une vascularite inflammatoire, multisystémique, caractérisée par la fréquence et la bénignité des manifestations cutanéomuqueuses et articulaires et la gravité des manifestations oculaires, neurologiques centrales, vasculaires et surtout artérielles et digestives [1]. Cette pathologie touche essentiellement l’homme (deux fois plus que la femme) entre 20 et 40 ans. Elle est fréquente en Extrême-Orient et sur le pourtour méditerranéen. Son diagnostic est clinique et repose sur des critères internationaux [1, 2]. C’est une maladie qui évolue par poussées parfois spontanément régressives et dont le traitement est essentiellement symptomatique du fait de nombreuses inconnues concernant son étiologie et sa physiopathologie [1]. La fréquence de l’atteinte cardiaque varie entre moins de 1 et 6% dans les séries cliniques et 16,5% dans une série autopsique [3]. Les trois tuniques cardiaques peuvent être touchées, avec péricardite, atteintes myocardiques, valvulaires, coronariennes et du tissu de conduction. La thrombose intracardiaque est très rare, une revue récente de la littérature faisait état de 25 observations rapportées. Cette complication survient généralement chez des hommes jeunes du bassin méditerranéen et du Moyen-Orient et prédomine dans les cavités droites du cœur [3].

## Patient et observation

**Figure 1:**
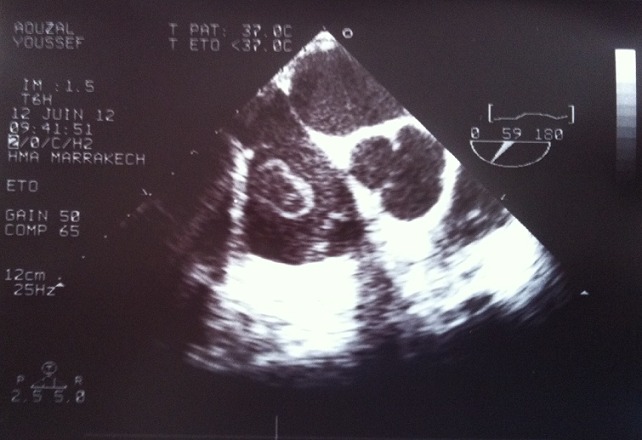
Image échocardiographique montrant la masse au niveau de l’oreillette et du ventricule droits

Nous rapportons l’observation clinique d’un jeune patient de 29 ans, ayant comme antécédents une aphtose bipolaire et qui a présenté depuis deux mois, un syndrome fébrile avec des hémoptysies, le tout évoluant dans un contexte d’altération de l’état général avec un amaigrissement chiffré à 10 kg au bout de deux mois. La radiographie pulmonaire a objectivé une pneumopathie traitée par une céphalosporine de 3^ème^ génération, sans nette amélioration. L’échocardiographie transthoracique a montré une masse au niveau de l’oreillette droite, de 13 mm de diamètre appendue au septum interauriculaire et s’abouchant dans la valve tricuspide et une masse du ventricule droit de 12 mm de diamètre et adhérente aux cordages de la tricuspide et à la partie moyenne du septum interventriculaire. L’échocardiographie transœsophagienne a confirmé le diagnostic de masse intracardiaque au niveau de l’oreillette et du ventricule droits (Figure 1). Le patient a été opéré par une stérnotomie médiane verticale, sous circulation extracorporelle, installé entre une canule artérielle au pied du tronc artériel brachiocéphalique et deux canules veineuses caves. L’atriotomie droite a mis en évidence une masse polylobée adhérente à la valvule du sinus coronaire, friable et facilement clivable et une autre masse du ventricule droit appendu aux cordages de la tricuspide et au septum interventriculaire. Les deux tumeurs ont été adressées pour examen anatomopathologique qui a objectivé l’aspect de thrombose pariétale. Les suites opératoires ont été marquées par la persistance de la fièvre et de l’asthénie, avec un syndrome inflammatoire biologique et des hémocultures négatives. Une échocardiographie de contrôle 8 jours après l’intervention a objectivé une récidive de la thrombose avec une petite masse de 6 mm de toit de l’oreillette droite et une masse apicale du ventricule droit de 7 mm. Un examen ophtalmologique à la recherche d’une uvéite était négatif. En outre un test de sensibilité cutanée est revenu positif. Par conséquent le diagnostic de maladie de Behçet a été retenu devant la récidive de la thrombose intracardiaque, les antécédents d’aphtose bipolaire et le test positif de sensibilité cutanée. Le patient a été mis sous corticothérapie (1mg/kg/24h) et colchicine (1mg/j) et anticoagulant (héparine sodique et relais par les antivitamines K) avec bonne évolution clinique.

## Discussion

Dans la littérature, l’atteinte cardiaque au cours de la MB la plus fréquente est la péricardite aiguë bénigne contemporaine d’une poussée inflammatoire de la maladie. Des myocardites survenant en l’absence de lésions des gros troncs coronaires ont été signalées et, enfin, de rares cas d’anévrismes ventriculaires spontanés ou après infarctus du myocarde ont été décrits [4]. Les thromboses intracardiaques sont exceptionnelles au cours de la MB; d’ailleurs sa découverte précède, dans la moitié des cas, le diagnostic de MB (le cas de notre observation). Depuis le premier cas autopsique rapporté par Buge et al. [5] en 1977, une quarantaine de cas de thromboses intracardiaques ont été rapportés au cours de la MB. Elles sont fréquemment associées à une fibrose endomyocardique et peuvent être également associées à une atteinte artérielle pulmonaire spécifique sous forme soit d’anévrismes pulmonaires soit d’atteinte parenchymateuse. Cela n’était pas le cas pour notre patient qui n’avait ni fibrose endomyocardique, ni anévrisme. L’étiologie de ces thromboses au cours de la MB est encore obscure, et le rôle des anticorps antiphospholipides dans cette maladie n’est pas établi [5]. L’association d’anévrismes artériels pulmonaires à la thrombose cardiaque de la MB comporte un risque d’hémoptysie et de décès par rupture des anévrysmes ainsi le risque hémorragique est majoré par la mise en route des anticoagulants devant la thrombose intracardiaque. La majorité des thromboses cardiaques de la MB touchent les cavités droites, les atteintes du cœur gauche étant plus rares. L’échographie permet quelques fois de suspecter un thrombus, mais l’aspect peut aussi évoquer un myxome ou une autre tumeur. L’IRM cardiaque, la scintigraphie aux plaquettes marquées à l’Indium 111 peuvent contribuer au diagnostic de thrombus cardiaque. Certains auteurs ont retrouvé chez des patients atteints de MB avec atteinte cardiovasculaire, des facteurs plasmatiques prothrombotiques (déficits en protéine C et S, augmentation du facteur VIII, homozygotie pour le facteur V Leiden ou mutation du gène de la prothrombine, anticorps antiphospholipides) [3, 6].

D’autres suggèrent des anomalies thrombogènes propres à la MB, comme la présence d’anticorps dirigés contre l’aénolase, une protéine cible à la surface des cellules endothéliales [7]. L’hyperhomocystéinémie modérée représente un facteur de risque de thrombose veineuse ou artérielle dans la population générale et s’associe aux carences en vitamines B9, B6, B12. Plusieurs publications notent des valeurs moyennes d’homocystéine plasmatique plus élevées chez des patients atteints d’une MB active par rapport aux groupes témoins, avec une association entre l’hyperhomocystéinémie et les atteintes vasculaires et ophtalmiques possibles [8, 9]. La mortalité des patients atteints de thrombose intracardiaque due à la MB est élevée, par hémoptysies massives spontanées secondaires aux anticoagulants utilisés ou postchirurgicales et plus rarement, embolie pulmonaire, rupture aortique ou infection favorisée par la corticothérapie [3]. Plusieurs auteurs ont rapporté la résolution de thrombus intracardiaque après traitement médical d’une MB. Les corticoïdes ont été employés seuls ou associés à la colchicine et/ou aux immunosuppresseurs (azathioprine, cyclophosphamide, ciclosporine) Les traitements par antivitamine K ou aspirine ont été utilisés. Leur emploi est délicat en cas d’hémoptysie associée [3]. Dincer et al. ont traité avec succès par fibrinolytiques une récidive thrombotique cardiaque postchirurgicale [10]. Dans notre cas, le traitement par corticoïdes, colchicine et anticoagulants (cible INR entre 3 et 4) a permis un contrôle de la MB. La chirurgie, réalisée en première intention dans des thromboses cardiaques inaugurant une MB, a un taux de complications et de décès élevé. Elle a été utilisée également pour le traitement d’une thrombose cardiaque récidivante malgré un traitement anticoagulant et immunosuppresseur. Chez notre patiente, l’intervention a permis de porter le diagnostic positif de thrombus.

## Conclusion

La thrombose intracardiaque est une complication rare de la MB, qui peut se présenter comme une tumeur intracardiaque. Sa découverte précède, dans la moitié des cas, le diagnostic de MB. La mortalité élevée peut être en rapport avec des complications post chirurgicales et/ou une atteinte associée des artères pulmonaires. La découverte d’une masse intracardiaque chez un sujet jeune doit faire évoquer le diagnostic de thrombus cardiaque et de MB, même en l’absence de facteur ethnique ou géographique prédisposant.
